# Items of Interest about the Pennsylvania Hospital

**Published:** 1886-02

**Authors:** 


### Items of Interest About the Pennsylvania Hospital.

There is a long chapter of interesting things about the Pennsylvania Hospital. It was there that Dr. Bond introduced the first course of clinical lectures for the instruction of medical students in this country. It started the first medical library, now one of the most valuable in the world. During the Revolution it was occupied by the British army, which despoiled it of its bedding, instruments, and everything of value. Stephen Girard's wife, who became insane, is buried in the hospital yard, and near by is the grave of Girard's only child, born while she was there. Had that child lived, the orphans of Philadelphia probably would not have become his heirs. It was there that the sufferers from the terrible epidemics of Philadelphia were treated. Out of the over-crowded condition of the place the Blockley Hospital and Almhouse came into existence. It furnished the nucleus for a medical museum. From its physicians and surgeons originated the greatest improvements in medical and surgical practice and appliances up to fifty years ago. It was the first fostering influence of pharmacy as an art in this country, and imported skilled chemists and compounders of medicine from abroad. It was the first medical institution in the country, if not in the world, to admit women as students to the clinical lectures, and out of this came the Women's Medical College of Philadelphia, which is sending women physicians as practitioners and missionaries all over the world. Many more things of importance and great public interest have had their rise in the Pennsylvania Hospital.

